# Adjuvant Treatment with Empagliflozin or Semaglutide Increases Endothelial Progenitor Cells in Subjects with Well-Controlled Type 1 Diabetes Mellitus

**DOI:** 10.3390/cimb47010054

**Published:** 2025-01-15

**Authors:** Maja Preložnik Navodnik, Katarina Reberšek, Katarina Klinar, Andrej Janež, Helena Podgornik

**Affiliations:** 1 Department of Angiology, Endocrinology and Rheumatology, General Hospital Celje, 3000 Celje, Slovenia; maja.navodnik-preloznik@sb-celje.si; 2 Department of Haematology, University Medical Centre Ljubljana, 1000 Ljubljana, Slovenia; katarina.rebersek@kclj.si (K.R.); katarina.klinar@kclj.si (K.K.); 3 Faculty of Pharmacy, University of Ljubljana, 1000 Ljubljana, Slovenia; 4 Department of Endocrinology, Diabetes and Metabolic Diseases, University Medical Centre Ljubljana, 1000 Ljubljana, Slovenia; andrej.janez@kclj.si; 5 Medical Faculty, University of Ljubljana, 1000 Ljubljana, Slovenia

**Keywords:** flow cytometry, type 1 diabetes mellitus, endothelial progenitor cells, circulating endothelial cells, adjuvant treatment, empagliflozin, semaglutide

## Abstract

Circulating endothelial cells (CECs) and endothelial progenitor cells (EPCs) are promising markers of vascular damage and endothelial regeneration potential. We focused on the detection of CECs and EPCs using flow cytometry with regard to analytical challenges and its suitability for routine testing. As part of a clinical validation, CECs and EPCs were measured in blood samples from 83 subjects with type 1 diabetes (T1DM), evaluating an adjuvant intervention with two different antidiabetic drugs, empagliflozin (N = 28) and semaglutide (N = 29). Both groups receiving adjuvant therapy were compared with the insulin-only group (N = 26) at two time points: before the start of therapy and after 12 weeks of adjuvant therapy. All three groups were comparable regarding demographic characteristics and concomitant risk factors. Absolute and relative endothelial cell count at baseline were low and comparable to those of healthy individuals. In the group receiving empagliflozin or semaglutide, a significant increase in EPC was observed after 12 weeks of treatment. We demonstrated that EPCs have the potential to serve as markers for monitoring the efficacy of adjuvant therapy in T1DM patients. However, before their implementation in clinical practice, the flow cytometry protocol for CEC and EPC identification and quantification must be standardized.

## 1. Introduction

Circulating endothelial cells (CECs) are mature cells released from damaged vascular walls [1]. Endothelial progenitor cells (EPCs), on the other hand, are bone marrow-derived cells involved in postnatal neovasculogenesis and vascular wall regeneration [2,3,4]. Therefore, CEC and EPC are promising markers of vascular damage and vascular regenerative potential [5,6]. Changes in CEC and EPC levels have been observed in many diseases associated with vascular dysfunction, such as infections, malignancies, cardiovascular, renal, and autoimmune diseases [6,7,8,9,10]. Therefore, the number of CECs and EPCs is a promising marker to determine disease prognosis and treatment response in various diseases [5,11]. Changes in the number of CECs and EPCs have also been described in diabetes mellitus (DM).

DM is characterized by vascular damage to the blood vessels and impaired vascular repair, resulting in an increased risk of cardiovascular disease (CVD). Factors for vascular damage in DM include elevated glucose levels, classic cardiovascular risk factors, arterial wall inflammation, and endothelial dysfunction [12,13]. While CECs are elevated in patients with type 1 DM (T1DM) and type 2 DM (T2DM), indicating endothelial damage, EPCs have been reported to be dysfunctional and their number reduced, leading to vascular damage and increased risk of CVD [12,14,15]. Metformin has been shown to have a potentially cardioprotective effect through EPCs and CECs in T1DM patients, independent of the hypoglycemic effect [16]. In addition, direct or indirect effects of some other antidiabetic drugs on EPC count or function have been proven [17].

The most commonly used method to identify and enumerate CECs and EPCs is polychromatic flow cytometry (PFC), as it allows for cell-by-cell analysis and the simultaneous determination of different cell markers, which is particularly important as no marker is completely specific to CEC or EPC. In addition, both cell populations are extremely rare in the peripheral blood, requiring a large events to be analyzed [18].

The immunophenotype of CECs and EPCs is still controversial and their identification is based on a combination of different markers, so an appropriate gating strategy is crucial. The most commonly used markers to identify CECs are CD31 and CD146. Some other markers, such as CD105 and CD141, can also be used [17,18,19,20]. The endothelial marker CD146 is also expressed in the lymphocyte subset and is used as an internal positive control [5].

Common markers used to identify EPCs include CD34, CD133, and vascular endothelial growth factor receptor 2 (VEGFR-2, also known as CD309/KDR). The distinction between CEC and EPC can be difficult due to an overlap in markers. CD34 is a hematopoietic stem cell marker expressed on both CECs and EPCs. On the other hand, CD133 is an immaturity marker and, unlike CD34, is not expressed in CECs. Therefore, CD133 could be a valuable factor in differentiating between CD133-positive EPCs and negative CECs. VEGFR-2, the most commonly used EPC marker, may also be present on hematopoietic stem cells (HSC). In addition, CD45 is used in the analysis of CECs and EPCs to exclude cells that are not of interest to the analysis. CECs and EPCs are reported to be CD45-negative and/or weakly positive [18]. Due to the rarity of these cell populations in the peripheral blood, additional strategies must be employed to exclude nonspecific events. An Fc blocking reagent, viability dyes, and nucleic acid dyes should be used to exclude dead cells and cells without a nucleus. It is also advisable to establish a dump channel (CD45, CD3, CD19, etc.) and to use isotypic control, to use fluorescence minus one (FMO), and to clean the cytometer between different sample analyses to prevent “carryover” [18,20]. Furthermore, indirect staining is not recommended [21].

Recently, we investigated the effects of antidiabetic drugs (empagliflozin (Boehringer Ingelheim International GmbH, Ingelheim am Rhein, Germany) and semaglutide (Novo Nordisk A/S, Novo Alle, Denmark) on metabolism and endothelial function as an add-on therapy alongside insulin in well-controlled subjects with T1DM [22]. We evaluated brachial artery blood flow, forearm blood flow, pulse wave velocity, and peripheral resistance as parameters for endothelial function and arterial stiffness. In a previously published paper, we confirmed the beneficial effect of both drugs and found that these drugs play a protective role in T1DM [22]. In addition to functional testing, endothelial function was also assessed via the enumeration of CECs and EPCs using flow cytometry. In this study, we focused on the detection of CECs and EPCs using flow cytometry with regard to analytical challenges and its suitability for routine testing.

## 2. Materials and Methods

### 2.1. Subjects

Eighty-three subjects with T1DM undergoing insulin treatment participated in a sub-study of the randomized, controlled, interventional Endothelium Dysfunction Assessment Study (ENDIS) on endothelial cells. The drugs tested were the sodium–glucose cotransporter 2 (SGLT-2) inhibitor empagliflozin and the glucagon-like peptide 1 (GLP-1) receptor agonist semaglutide [22]. Subjects enrolled in the study were randomized into three comparable groups based on their demographic characteristics and concomitant risk factors. In the first group, patients received adjuvant therapy with empagliflozin, patients in the second group received adjuvant therapy with semaglutide, and patients in the control group did not receive adjuvant therapy. All patients were naïve to both the drugs used and metformin to avoid potential confounding effects on EPC and CEC number [16]. In addition, patients with a history of any manifested atherosclerotic disease, renal insufficiency (creatinine clearance < 60 mL/min), known active malignancies, chronic systemic connective tissue diseases or chronic wounds, poorly controlled diabetes (HbA1c > 9.0%), or a body mass index < 20 were excluded. In order to limit possible influences on the EPC count, all patients included in the study were asked to refrain from taking vitamin supplements and intensive physical activity before the two study visits and to appear healthy and without cold symptoms. In addition, the women were asked to present in the same period of their menstrual cycle [7,23,24,25,26,27]. The basic characteristics of the patients involved in our study are shown in Table 1.

Peripheral blood samples from 10 patients collected prior to stem cell harvesting were used for analytical verification of the method, specifically, to compare the HSC count obtained using the established protocol for EPC and CEC enumeration with the HSC count obtained by the International Society of Hematotherapy and Graft Engineering (ISHAGE) protocol and to assess imprecision.

The study was approved by the Slovenian National Medical Ethics Committee (No. 0120-63/2020/10). In addition, all participants gave their written informed consent according to the Declaration of Helsinki. The study was registered at Clinicaltrials.gov (NCT05857085).

### 2.2. Sample Collection and Analysis

Peripheral blood was collected in 10 mL EDTA tubes. To avoid the effects of vascular damage caused by venipuncture, which can lead to an increase in CEC count [28], the first collection tube was used for other analyses. Samples were collected twice, at baseline—before the initiation of adjuvant treatment (V1) and after 12 weeks of treatment (V2).

All samples were processed within 2–4 h after collection to minimize the effects of CEC and EPC instability on the analytical results. Blood samples were transported at room temperature. The WBC count was determined using the Sysmex XN-1000 hematology analyzer (Sysmex Corporation, Kobe, Japan) and used to calculate the absolute CEC and EPC count. The protocol for EPC and CEC identification using flow cytometry was established according to Lanuti et al. [19]. Peripheral blood was subjected to erythrocyte lysis with NH_4_Cl. After incubation at room temperature for 10 min and centrifugation (5 min at 522× *g*, room temperature), the cells were resuspended in 2 mL of PBS and washed twice. The absolute WBC count was adjusted to 45 × 10^9^/L with PBS and 100 µL of the cell suspension was stained with 3 µL CD133-Vio^®^Bright FITC (Miltenyi Biotec, Cologne, Germany), 5 µL VEGFR-2-PE (RD Systems, Minneapolis, MN, USA), 5 µL CD146-PC7 (BD Biosciences, Franklin Lakes, NJ, USA), 5 µL CD34-APC (BD Biosciences, Franklin Lakes, NJ, USA), 5 µL CD45-KrO (Beckman Coulter, Brea, CA, USA), 5 µL 7-AAD (Beckman Coulter, Brea, CA, USA), and 5 µL of 133 µM Syto40 (Thermo Fisher Scientific, Waltham, MA, USA). In the second tube, 100 µL of the cell suspension was stained with the corresponding isotype controls. After 30 min incubation in the dark at room temperature, the stained cells were washed with PBS, centrifuged, and resuspended in 500 µL PBS. Immediately after washing, the acquisition was carried out using the flow cytometer (Navios, Beckman Coulter, Brea, CA, USA). The optical alignment and fluidic system were checked daily prior to sample acquisition using Flow-Check Pro Fluorospheres (Beckman Coulter, Brea, CA, USA) and standardization was performed weekly using Flow-Set Pro Fluorospheres (Beckman Coulter, Brea, CA, USA) according to the manufacturer’s instructions. Kaluza Analysis Software (version 2.1, Beckman Coulter, Brea, CA, USA) was used for data analysis.

All antibodies and dyes were titrated under assay conditions to obtain optimal dilutions. Compensation was performed using single-stained samples. The specificity of the binding of antibodies (anti-CD146, VEGFR-2, and CD133) was tested with isotype-matched controls at the same concentration and from the same manufacturer as the respective antibody. In addition to the isotype controls used in each analysis, an FMO was performed at the time of assay verification to assess non-specific fluorescence.

Five samples were analyzed in triplicate in a single run to determine the imprecision of the test. To avoid the influence of the instability of CECs and EPCs, the imprecision was determined using the HSC population in the samples. The HSC count determined according to our protocol was compared with the HSC count determined according to the ISHAGE protocol in 10 samples [29]. Stem kit reagents (Beckman Coulter, Brea, California, USA) were used for counting HSCs according to the ISHAGE protocol. Intra-assay CV was 5.34%, below the acceptable imprecision limit (10%). In addition, a very strong correlation (Spearman correlation coefficient 0.988) and small differences between the HSC count determined with our protocol and the ISHAGE protocol were confirmed.

Using the verified protocol, CECs and EPCs were analyzed in samples from 83 patients. For each sample, more than 1,000,000 WBC (an average, 3.7 × 10^6^) were analyzed.

### 2.3. Gating Strategy

Given that CECs exhibit mononuclear cell morphology, the first step was to gate the lymphocyte–monocyte population in the SS/FS plot, followed by the exclusion of dead cells based on 7-AAD positivity in the 7AAD/SS plot and cells without nucleic acids based on Syto40 negativity in the Syto40/SS plot. Thereafter, the surface antigen expression was analyzed in selected cells. CEC were identified as CD45-negative or weakly positive in the CD45/SS plot, while CD45 also enabled the explicit recognition of the lymphocyte subset, which served as an internal positive control for CD146. The recognition of lymphocytes based solely on cell morphological features in the FS/SS plot is challenging because of the large number of cells analyzed, resulting in less distinct boundaries between different cell populations. Consequently, CD146- and VEGFR-2-positive and -negative populations in the CD146/VEGFR-2 plot were demarcated based on lymphocytes recognized in the CD45/SS plot. CD45-negative or weakly positive cells were subsequently analyzed for CD34 expression. Only CD34-positive cells were subsequently gated in the CD34/SS plot; following this, CD146-positive cells were gated in the CD146/VEGFR-2 plot. Finally, of these cells, only those that were CD133-negative in the CD34/CD133 plot were identified as CECs.

**Figure 1:**
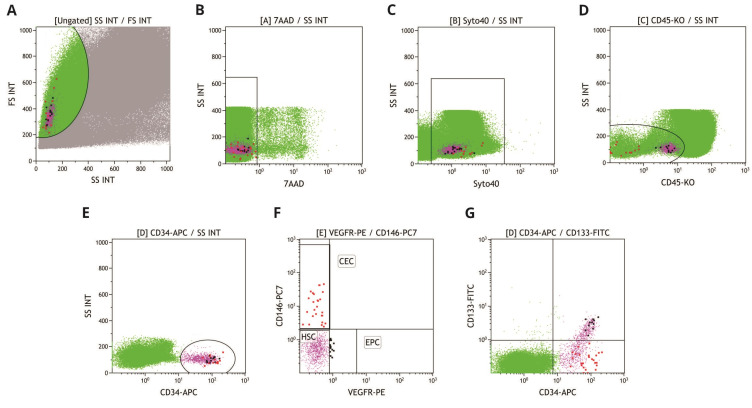
Gating strategy for the identification of CECs and EPCs in the peripheral blood. (**A**) gating of mononuclear cells in forward scatter (FS INT) versus side scatter (SS INT); (**B**) gating of live cells (7-AAD); (**C**) gating of cells with nucleic acids (Syto40+); (**D**) gating of CD45−/weakly+; (**E**) gating of CD34+ cells; (**F**) CEC (CD146+, red) and EPC (VEGFR-2+, black) identification; (**G**) additional differentiation between CECs (CD133−, red) and EPCs (CD133+, black).

For EPCs, which also exhibit a mononuclear cell morphology, the lymphocyte–monocyte population was gated in the SS/FS plot, in addition to excluding dead cells and cells without nucleic acids as in the CEC analysis. Subsequently, only weakly CD45-positive cells were gated in the CD45/SS plot. Further only CD34-positive cells in the CD34/SS plot were analyzed for VEGFR-2 positivity in the CD146/VEGFR-2 plot. Finally, only CD133-positive cells were considered EPCs in the CD133/CD34 plot. Figure 1 presents the gating strategy used for the identification of CECs and EPCs.

For HSCs, which also exhibit a mononuclear cell morphology, the lymphocyte–monocyte population was gated in the SS/FS plot, in addition to the exclusion of dead cells and cells without nucleic acids, as in the CEC analysis. Subsequently, only weakly CD45-positive cells and CD34-positive cells were gated in the CD45/SS and CD34/SS plots.

As CD45 is a pan-leukocyte marker, WBCs were identified as CD45-positive cells gated in the CD45/SS plot.

### 2.4. Relative and Absolute Cell Count Enumeration

The relative cell count of EPCs and CECs was expressed as a percentage of the total leukocyte count determined via flow cytometry. Absolute cell count was calculated by dual platform, using relative CEC or EPC count, as described above, and the WBC count determined via hematology analyzer (Sysmex XN-1000, Sysmex Corporation, Kobe, Japan).

### 2.5. Statistics

The software package MedCalc^®^ Statistical Software Version 20.027 (MedCalc Software Ltd., Ostend, Belgium) was used for the statistical analysis. The Shapiro–Wilk test was used to assess the normality distribution. Imprecision was determined using the coefficient of variation (CV). Correlation was tested using Spearman’s rank correlation. CEC and EPC changes between the two visits (V1 vs. V2) were tested using the Wilcoxon signed rank test. Differences between groups were tested using an independent sample Kruskal–Wallis test. All statistical tests used were two-sided and statistical significance was assumed at *p* < 0.05.

## 3. Results

The enumeration of CEC and EPC was a secondary exploratory analysis of the interventional Endothelium Dysfunction Assessment Study (ENDIS). Flow cytometry was performed on samples from a subset of 83 patients with well-controlled T1DM who were randomized into three comparable groups. In the first group, patients received adjuvant therapy with empagliflozin, patients in the second group received adjuvant therapy with semaglutide, and patients in the control group received no adjuvant therapy. The endothelial cell analysis was performed before the start of treatment (V1) and repeated after 12 weeks of adjuvant therapy (V2). The baseline characteristics of the patients involved in our study (Table 1), their demographic characteristics, and concomitant risk factors were not statistically different from previously published data for the entire cohort of 92 patients [22]. The age of the participants did not differ significantly between groups (*p* = 0.6759), limiting the concern that age may be a contributor to response to adjuvant treatment. CECs were detected in all samples at V1, with a median of 10.24/mL (ranging from 1.46/mL to 99.78/mL). On the other hand, EPCs were detected in only 60% of the samples analyzed at V1, with a median of 1.95/mL (ranging from 0.0/mL to 36.25/mL). Table 1 shows the results of CEC, EPC, HSC and white blood cell (WBC) enumeration for all three groups at two different time points. There were no significant differences between groups in all tested parameters.

In the group receiving empagliflozin, a significant increase in relative (*p* = 0.004) and absolute (*p* = 0.002) EPC count was observed after 12 weeks of treatment, while a decrease in relative (*p* = 0.265) and absolute (*p* = 0.350) CEC count was not significant (Table 2).

**Figure 2:**
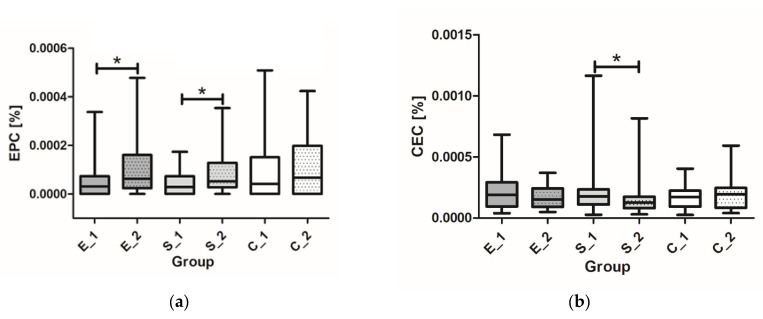
Differences in relative EPC (**a**) and CEC counts (**b**) for time point V1 vs. time point V2 within groups. (E_1 empagliflozin group at time point V1, E_2 empagliflozin group at time point V2, S_1 semaglutide group at time point V1, S_2 semaglutide group at time point V2, C_1 control group at time point V1, and C_2 control group at time point V2). * *p* < 0.05 significance level.

A 12-week treatment with semaglutide led to a significant increase in relative (*p* = 0.001) and absolute (*p* = 0.001) EPC count (Table 2). In addition, a significant decrease in relative (*p* = 0.014) and absolute (*p* = 0.018) CEC count was observed with semaglutide during the same treatment period. In the control group, as expected, no significant changes were observed in the relative (*p* = 0.983) and absolute (*p* = 0.534) EPC count or in the relative (*p* = 0.809) and absolute (*p* = 0.657) CEC count (Table 2, Figure 2). As expected, there were no significant differences in absolute and relative HSC and WBC counts after 12 weeks of treatment in all groups (Table 2).

## 4. Discussion

In addition to elevated glucose levels, classic cardiovascular risk factors, and inflammation of the arterial walls, endothelial dysfunction also increases vascular impairment and CVD risk in DM [12,13]. Changes in endothelial cell levels are a potential marker of endothelial impairment in DM patients; CECs are elevated, indicating endothelial damage, while EPCs are dysfunctional and reduced [12,14,15].

In this study, we focused on the detection of CECs and EPCs using flow cytometry with regard to their challenges and their suitability as part of clinical validation. CECs and EPCs were enumerated in T1DM patients treated with empagliflozin and semaglutide. CECs and EPCs are rare cell populations in the peripheral blood with a complex immunophenotype, which makes their identification and quantification via flow cytometry challenging. While a standardized protocol for CEC detection was previously proposed [30], there is currently no consensus regarding the immunophenotype of EPCs. Various studies have used different combinations of markers, particularly CD34, CD133, and VEGFR-2, to identify them [20,21]. The detection and enumeration of CECs and EPCs are further complicated by the lack of available standards or reference materials.

When deciding on the positive or negative markers that should be included in the test panel for CEC and EPC detection, their number is dependent on the number of colors that can be analyzed simultaneously using flow cytometry. Increasing the number of markers would thus require the use of an additional test tube, increasing the acquisition time per sample and affecting the reliability of the results due to instability. In addition, a larger volume of blood is required to ensure a sufficient number of cells are available for analysis. We generally followed the previously proposed protocol but excluded CD31 from the panel [20]. Additional approaches, such as the use of isotype controls, viability, and nucleic acid stains, were adopted to eliminate nonspecific events. The seven-color PFC protocol with a stepwise gating strategy allowed us to distinguish rare CEC and EPC populations from WBCs as well as from debris. CECs were recognized as live nucleated cells that were CD34-positive, CD45-negative or weakly positive, CD133-negative, and CD146-positive. EPCs were identified as live nucleated cells positive for CD34, weakly positive for CD45, positive for CD133, and positive for VEGFR-2. The results were expressed both as a relative (number of CECs and EPCs per all WBCs analyzed) and as an absolute count (number of CECs and EPCs per mL of blood). For the reasons mentioned above, which limit the number of markers that can used, the markers that would identify the source of the EPCs or CECs were not included. As circulatory biomarkers only indicate tissue status and do not confirm disease, different markers/methods should be used to corroborate disease status [31]. Although a correlation between CECs and some plasma biochemical markers, such as the von Willebrand factor, soluble E-selectin, and tissue factor has been demonstrated, these markers are not fully specific to vascular damage. Consequently, they may not confirm the findings obtained in the tissue, whereas CECs originate directly from the endothelium [6,32,33].

Another open question is whether EPC and CEC data should be presented as absolute or relative counts, as both parameters can be influenced by physiological or pathological conditions. Hemoconcentration and hemodilution, which are common in various cardiovascular diseases, may influence the absolute count determination, while the relative count may be influenced by leukocytosis or leukopenia [21]. Since both the absolute and the relative count have been used in previous studies, we decided to report both to better compare the populations of CECs and EPCs with previously published results.

Due to the uncertainties mentioned above regarding the immunophenotype of the endothelial cells and the technical problems associated with their enumeration, a direct comparison of the results of different studies is difficult. Our results are comparable to the absolute CEC count with a similar immunophenotype determined by Lanuti et al. and Almici et al. in healthy controls [30,34]. Although a higher absolute count would be expected in patients with T1DM, only patients with controlled T1DM and without severe endothelial complications were included in our study (Table 1), which may explain why the results are comparable to the healthy population and also confirms the reliability of our method.

It is even more difficult to compare the results for EPCs because different studies used different combinations of markers, particularly CD34, CD133, and VEGFR-2 [20,21]. In addition, the fluorescence intensity of VEGFR-2 is low compared to the isotype control, which could lead to a falsely lower EPC count if the cut-off between positive and negative populations is set according to the lymphocyte subset. On the other hand, if the cut-off is set according to the isotype control, this could lead to falsely elevated results, as the HSC subset could also be included. Therefore, we decided to use the lymphocyte subset to distinguish between positive and negative populations for VEGFR-2. Another problem with EPC enumeration is that visual assessment of the population which is based onlow antigen expression may lead to subjective assessments dependent on the investigator’s expertise.

We observed an effect of adjuvant treatment on endothelial function after 12 weeks of treatment with empagliflozin and semaglutide, with an increase in EPCs compared to both the baseline and the control group (Table 2, Figure 2). During the 12-week period CECs were decreased only in the semaglutide-treated group. This is likely due to the similar characteristics of the patients to the healthy population which explains the relatively low baseline CEC values found in the group. Previous studies have shown that GLP-1 stimulates the proliferation and differentiation of EPCs in vitro [35]. In another study, an increase in EPCs was observed in patients with T2DM after treatment with a GLP-1 analog. While no short-term effects (12-week treatment) on the number of CECs and EPCs were detected in empagliflozin-treated patients with T2DM, an increase in EPCs was observed with long-term treatment [36]. We found a significant increase in EPCs in patients with T1DM after 12 weeks of adjuvant therapy with empagliflozin or semaglutide and a significant decrease in CECs in semaglutide-treated patients. Our results are consistent with the above studies, as both SGLT-2 inhibitors and GLP-1 analogs have been shown to reduce the risk of major cardiovascular events [37], in addition to improving endothelial function [38]. However, a direct effect of SGLT-2 inhibitors on the mobilization of EPCs has not been demonstrated [39] and the use of SGLT-2 inhibitors and GLP-1 analogs as an adjuvant therapy for T1DM is currently not approved. Similarly, it cannot be determined whether the increase in EPCs in our cohort is due to the direct effect of the tested drugs on their mobilization or merely to better glycemic control.

The ENDIS already confirmed a positive effect of adjuvant therapy with SGLT-2 inhibitors and GLP-1 analogs on endothelial function in T1DM patients based on various functional tests [22]. The present study confirmed this through a noninvasive laboratory test, namely a flow cytometric evaluation of endothelial cells. There are, however, a number of limitations that should be considered. Firstly, the enumeration of EPCs and CECs is cumbersome; therefore, for the routine use of this test, a standardized analytical approach is required, including the careful selection and combination of markers, an analytical verification of the method, and an efficient gating strategy. Secondly, due to the rarity of these cells in the peripheral blood, a high number of events must be analyzed to obtain a reliable count of CECs and EPCs, which prolongs the acquisition time and consequently limits the number of samples that can be analyzed simultaneously. In addition, no consensus has yet been reached on the most appropriate immunophenotype of CECs and especially EPCs. However, on the basis of the observed differences in CEC and EPC counts in the therapeutic groups, CEC and EPC determination using flow cytometry has the potential for integration into routine clinical practice to monitor the efficacy of adjuvant therapy in T1DM patients. Finally, it is difficult to distinguish to what extent the improvements in EPC and CEC values are due to glycemic or body weight control via adjuvant therapy. Considering the complexity of DM, many other confounding factors should be taken into consideration prior to its implementation into clinical practice. In addition, the long-term effect of the tested drugs on CEC and EPC count and other clinical characteristics should also be assessed in future research.

## 5. Conclusions

A positive effect of adjuvant treatment on endothelial function with empagliflozin and semaglutide in patients with well-controlled T1DM was demonstrated as an increase in EPCs. EPCs have the potential to serve as markers for monitoring the efficacy of adjuvant therapy in T1DM patients. However, prior to its implementation into clinical practice, a standardization of the flow cytometry protocol for CEC and EPC identification and quantification is required. In addition, regarding the complexity of DM, many other confounding factors should be taken into consideration in future research.

## Figures and Tables

**Table 1:** Characteristics of T1DM patients.

Parameters	Empagliflozin (n = 28)	Semaglutide (n = 29)	Control (n = 26)
Median age (range)	49 (20–66)	49 (24–65)	44.5 (23–69)
Young adults (%) Middle aged adults (%) Elders (%)	5 (18) 23 (82) 0 (0)	4 (14) 24 (83) 1 (3)	8 (31) 15 (58) 3 (11)
Gender (males/females)	17/11	18/11	15/11
Median T1DM duration (years), (IQR)	18.0 (15.8–31.3)	24 (14–29)	20.5 (9.5–25)
Median HbA1c (%), (IQR)	8.1 (7.5–8.5)	7.7 (6.7–8.1)	6.9 (6.4–7.8)
Median glucose (mmol/L), (IQR)	9.3 (7.3–10.8)	7.5 (5.6–9.6)	7.2 (5.3–9.2)

IQR—interquartile range, young adults (<40 years), middle aged adults (40–65 years), and elders (>65 years).

**Table 2:** Relative and absolute CEC and EPC counts before the start of treatment (V1) and after 12 weeks (V2) of treatment for the three tested groups.

	Empagliflozin (n = 28)	*p* *	Semaglutide (n = 29)	*p* *	Control (n = 26)	*p* *	*p* ^#^
V1 CEC (% × 10^−4^)	1.90 (1.00–2.76)	0.265	1.78 (1.13–2.33)	**0.014**	1.72 (0.95–2.18)	0.809	*p* = 0.733
V2 CEC (% × 10^−4^)	1.52 (0.94–2.39)		1.26 (0.81–1.72)		1.94 (0.4, 5.9)		*p* = 0.467
V1 CEC (/mL)	9.32 (6.58–15.17)	0.350	10.82 (6.82–15.42)	**0.018**	9.99 (7.70–12.91)	0.657	*p* = 0.909
V2 CEC (/mL)	8.71 (6.37–12.75)		7.92 (6.29–11.24)		9.60 (4.77–13.94)		*p* = 0.884
V1 EPC (% × 10^−4^)	0.31 (0.0–0.72)	**0.004**	0.28 (0.0–0.71)	**0.001**	0.42 (0.0–1.43)	0.983	*p* = 0.485
V2 EPC (% × 10^−4^)	0.63 (0.23–1.60)		0.52 (0.28–1.25)		0.67 (0.0–1.93)		*p* = 0.915
V1 EPC (/mL)	2.08 (0.0–3.83)	**0.002**	1.63 (0.0–3.81)	**0.001**	2.34 (0.0–10.04)	0.534	*p* = 0.486
V2 EPC (/mL)	3.82 (1.48–10.15)		3.65 (1.53–8.22)		3.32 (0.0–9.14)		*p* = 0.871
V1 HSC (%)	0.03 (0.02–0.04)	0.776	0.02 (0.02–0.04)	0.939	0.03 (0.02–0.04)	0.835	*p* = 0.9645
V2 HSC (%)	0.03 (0.02–0.03)		0.03 (0.02–0.03)		0.03 (0.02–0.04)		*p* = 0.7105
V1 HSC (/mL)	1.63 (1.13–2.21)	0.955	1.59 (1.26–2.43)	0.758	1.83 (1.05–2.39)	0.710	*p* = 0.9617
V2 HSC (/mL)	1.62 (1.15–2.32)		1.80 (1.36–2.25)		1.71 (1.27–2.20)		*p* = 0.8207
V1 WBC (×10^9^/L)	6.09 (5.05–6.73)	0.974	6.06 (5.08–7.1)	0.655	5.81 (5.27–7.00)	0.365	*p* = 0.8278
V2 WBC (×10^9^/L)	5.78 (4.89–7.33)		6.18 (5.3–7.75)		5.83 (4.82–6.48)		*p* = 0.1926

* Wilcoxon signed rank test; ^#^ independent samples Kruskal–Wallis Test, *p* < 0.05 significance level. Bold: values < 0.05.

## Data Availability

The raw data supporting the conclusions of this article will be made available by the authors on request.

## References

[1] Kraan J., Strijbos M.H., Sieuwerts A.M., Foekens J.A., Bakker M.A.D., Verhoef C., Sleijfer S., Gratama J.W. (2012). A new approach for rapid and reliable enumeration of circulating endothelial cells in patients. J. Thromb. Haemost..

[2] Asahara T., Masuda H., Takahashi T., Kalka C., Pastore C., Silver M., Kearne M., Magner M., Isner J.M. (1999). Bone Marrow Origin of Endothelial Progenitor Cells Responsible for Postnatal Vasculogenesis in Physiological and Pathological Neovascularization. Circ. Res..

[3] Asahara T., Murohara T., Sullivan A., Silver M., van der Zee R., Li T., Witzenbichler B., Schatteman G., Isner J.M. (1997). Isolation of Putative Progenitor Endothelial Cells for Angiogenesis. Science.

[4] Zampetaki A., Kirton J.P., Xu Q. (2008). Vascular repair by endothelial progenitor cells. Cardiovasc. Res..

[5] Řádek M., Babuňková E., Špaček M., Kvasnička T., Kvasnička J. (2019). Determination of Circulating Endothelial Cells and Endothelial Progenitor Cells Using Multicolor Flow Cytometry in Patients with Thrombophilia. Acta Haematol..

[6] Zhang K., Yin F., Lin L. (2014). Circulating Endothelial Cells and Chronic Kidney Disease. BioMed Res. Int..

[7] Guervilly C., Burtey S., Sabatier F., Cauchois R., Lano G., Abdili E., Daviet F., Arnaud L., Brunet P., Hraiech S. (2020). Circulating Endothelial Cells as a Marker of Endothelial Injury in Severe COVID-19. J. Infect. Dis..

[8] Danova M., Comolli G., Manzoni M., Torchio M., Mazzini G. (2016). Flow cytometric analysis of circulating endothelial cells and endothelial progenitors for clinical purposes in oncology: A critical evaluation. Mol. Clin. Oncol..

[9] Farinacci M., Krahn T., Dinh W., Volk H.D., Düngen H.D., Wagner J., Konen T., von Ahsen O. (2019). Circulating endothelial cells as biomarker for cardiovascular diseases. Res. Pract. Thromb. Haemost..

[10] Di Martino M.L., Frau A., Losa F., Muggianu E., Mura M.N., Rotta G., Scotti L., Marongiu F. (2021). Role of circulating endothelial cells in assessing the severity of systemic sclerosis and predicting its clinical worsening. Sci. Rep..

[11] Triggle C.R., Ding H., Marei I., Anderson T.J., Hollenberg M.D. (2020). Why the endothelium? The endothelium as a target to reduce diabetes-associated vascular disease. Can. J. Physiol. Pharmacol..

[12] Arcangeli A., Lastraioli E., Piccini B., D’amico M., Lenzi L., Pillozzi S., Calabrese M., Toni S., Arcangeli A. (2017). Circulating Endothelial Progenitor Cells in Type 1 Diabetic Patients: Relation with Patients’ Age and Disease Duration. Front. Endocrinol..

[13] Zahran A.M., Mohamed I.L., El Asheer O.M., Tamer D.M., Abo-Elela M.G.M., Abdel-Rahim M.H., El-Badawy O.H.B., Elsayh K.I. (2019). Circulating Endothelial Cells, Circulating Endothelial Progenitor Cells, and Circulating Microparticles in Type 1 Diabetes Mellitus. Clin. Appl. Thromb..

[14] Asicioglu E., Yavuz D.G., Koc M., Ozben B., Yazici D., Deyneli O., Akalin S. (2010). Circulating endothelial cells are elevated in patients with type 1 diabetes mellitus. Eur. J. Endocrinol..

[15] McClung J.A., Naseer N., Saleem M., Rossi G.P., Weiss M.B., Abraham N.G., Kappas A. (2005). Circulating endothelial cells are elevated in patients with type 2 diabetes mellitus independently of HbA(1)c. Diabetologia.

[16] Ahmed F.W., Rider R., Glanville M., Narayanan K., Razvi S., Weaver J.U. (2016). Metformin improves circulating endothelial cells and endothelial progenitor cells in type 1 diabetes: MERIT study. Cardiovasc. Diabetol..

[17] Altabas V., Radošević J.M., Špoljarec L., Uremović S., Bulum T. (2023). The Impact of Modern Anti-Diabetic Treatment on Endothelial Progenitor Cells. Biomedicines.

[18] Khan S.S., Solomon M.A., McCoy J.P. (2005). Detection of circulating endothelial cells and endothelial progenitor cells by flow cytometry. Cytom. B Clin. Cytom..

[19] Duda D.G., Cohen K.S., Scadden D.T., Jain R.K. (2007). A protocol for phenotypic detection and enumeration of circulating endothelial cells and circulating progenitor cells in human blood. Nat. Protoc..

[20] Lanuti P., Rotta G., Almici C., Avvisati G., Budillon A., Doretto P., Malara N., Marini M., Neva A., Simeone P. (2016). Endothelial progenitor cells, defined by the simultaneous surface expression of VEGFR2 and CD133, are not detectable in healthy peripheral and cord blood. Cytom. Part A J. Int. Soc. Anal. Cytol..

[21] Fadini G.P., Baesso I., Albiero M., Sartore S., Agostini C., Avogaro A. (2008). Technical notes on endothelial progenitor cells: Ways to escape from the knowledge plateau. Atherosclerosis.

[22] Navodnik M.P., Janež A., Žuran I. (2023). The Effect of Additional Treatment with Empagliflozin or Semaglutide on Endothelial Function and Arterial Stiffness in Subjects with Type 1 Diabetes Mellitus—ENDIS Study. Pharmaceutics.

[23] Sen A., Vincent V., Thakkar H., Abraham R., Ramakrishnan L. (2022). Beneficial Role of Vitamin D on Endothelial Progenitor Cells (EPCs) in Cardiovascular Diseases. J. Lipid Atheroscler..

[24] Wong C., Qiuwaxi J., Chen H., Li S., Chan H., Tam S., Shu X., Lau C., Kwong Y., Tse H. (2008). Daily intake of thiamine correlates with the circulating level of endothelial progenitor cells and the endothelial function in patients with type II diabetes. Mol. Nutr. Food Res..

[25] Volaklis K.A., Tokmakidis S.P., Halle M. (2013). Acute and chronic effects of exercise on circulating endothelial progenitor cells in healthy and diseased patients. Clin. Res. Cardiol..

[26] Robb A., Mills N., Smith I., Short A., Tura-Ceide O., Barclay G., Blomberg A., Critchley H., Newby D., Denison F. (2009). Influence of menstrual cycle on circulating endothelial progenitor cells. Hum. Reprod..

[27] Tanaka S., Ueno T., Sato F., Chigusa Y., Kawaguchi-Sakita N., Kawashima M., Fujisawa N., Yoshimura K., Teramukai S., Fujiwara H. (2012). Alterations of Circulating Endothelial Cell and Endothelial Progenitor Cell Counts around the Ovulation. J. Clin. Endocrinol. Metab..

[28] Goon P.K.Y., Lip G.Y.H., Boos C.J., Stonelake P.S., Blann A.D. (2006). Circulating Endothelial Cells, Endothelial Progenitor Cells, and Endothelial Microparticles in Cancer. Neoplasia.

[29] Keeney M., Chin-Yee I., Weir K., Popma J., Nayar R., Sutherland D.R. (1998). Single platform flow cytometric absolute CD34+ cell counts based on the ISHAGE guidelines. International Society of Hematotherapy and Graft Engineering. Cytometry.

[30] Lanuti P., Simeone P., Rotta G., Almici C., Avvisati G., Azzaro R., Bologna G., Budillon A., Di Cerbo M., Di Gennaro E. (2018). A standardized flow cytometry network study for the assessment of circulating endothelial cell physiological ranges. Sci. Rep..

[31] Markova V., Bogdanov L., Velikanova E., Kanonykina A., Frolov A., Shishkova D., Lazebnaya A., Kutikhin A. (2023). Endothelial Cell Markers Are Inferior to Vascular Smooth Muscle Cells Markers in Staining Vasa Vasorum and Are Non-Specific for Distinct Endothelial Cell Lineages in Clinical Samples. Int. J. Mol. Sci..

[32] Lee K.W., Blann A.D., Lip G.Y. (2006). Inter-relationships of indices of endothelial damage/dysfunction [circulating endothelial cells, von Willebrand factor and flow-mediated dilatation] to tissue factor and interleukin-6 in acute coronary syndromes. Int. J. Cardiol..

[33] Boos C.J., Balakrishnan B., Blann A.D., Lip G.Y.H. (2008). The relationship of circulating endothelial cells to plasma indices of endothelial damage/dysfunction and apoptosis in acute coronary syndromes: Implications for prognosis. J. Thromb. Haemost..

[34] Almici C., Neva A., Skert C., Bruno B., Verardi R., Di Palma A., Bianchetti A., Braga S., Piovani G., Cancelli V. (2019). Counting circulating endothelial cells in allo-HSCT: An ad hoc designed polychromatic flowcytometry-based panel versus the CellSearch System. Sci. Rep..

[35] Xie X.Y., Mo Z.H., Chen K., He H.H., Xie Y.H. (2011). Glucagon-like Peptide-1 improves proliferation and differentiation of endothelial progenitor cells via upregulating VEGF generation. Med. Sci. Monit. Int. Med. J. Exp. Clin. Res..

[36] De Ciuceis C., Agabiti-Rosei C., Rossini C., Caletti S., Coschignano M.A., Ferrari-Toninelli G., Ragni G., Cappelli C., Cerudelli B., Airò P. (2018). Microvascular Density and Circulating Endothelial Progenitor Cells Before and After Treatment with Incretin Mimetics in Diabetic Patients. High Blood Press. Cardiovasc. Prev..

[37] Giugliano D., Scappaticcio L., Longo M., Bellastella G., Esposito K. (2021). GLP-1 receptor agonists vs. SGLT-2 inhibitors: The gap seems to be leveling off. Cardiovasc. Diabetol..

[38] Wang Y., Yao M., Wang J., Liu H., Zhang X., Zhao L., Hu X., Guan H., Lyu Z. (2022). Effects of Antidiabetic Drugs on Endothelial Function in Patients With Type 2 Diabetes Mellitus: A Bayesian Network Meta-Analysis. Front. Endocrinol..

[39] Bonora B.M., Cappellari R., Albiero M., Avogaro A., Fadini G.P. (2018). Effects of SGLV2 Inhibitors on Circulating Stem and Progenitor Cells in Patients With Type 2 Diabetes. J. Clin. Endocrinol. Metab..

